# Preliminary Efficacy of an mHealth Mindfulness Intervention With Caregivers of Older Adults With MCI or Dementia

**DOI:** 10.1093/geroni/igab046.156

**Published:** 2021-12-17

**Authors:** Elissa Kozlov, Danielle Llaneza, XinQi Dong, Paul Duberstein

**Affiliations:** 1 Rutgers University, Piscataway, New Jersey, United States; 2 Rutgers University, New Brunswick, New Jersey, United States; 3 Rutgers University, Rutgers Institute for Health, New Jersey, United States; 4 Rutgers University School of Public Health, Piscataway, New Jersey, United States

## Abstract

Decades of research have documented the negative effects of caregiving on unpaid caregivers. Caregivers are more likely to suffer from high levels of stress and anxiety, and caregivers of older adults with dementia are at especially high risk. Mindfulness Therapy (MT) is a promising, non-pharmacological technique with proven efficacy and effectiveness in managing stress and anxiety in diverse populations. Mindfulness Coach is an m-health delivered mindfulness therapy intervention developed by the Veterans Affairs National Center for PTSD. The objective of this paper is to report the preliminary efficacy of an 8-week pilot trial of mHealth-delivered mindfulness therapy to alleviate anxiety and caregiver stress in caregivers of persons with dementia. Sixty caregivers of patients with mild cognitive impairment or dementia were recruited to participate in this single group pre-post design study. After receiving an orientation to using the app, participants were instructed to use the app daily to learn about and practice mindfulness skills. At the end of the 8 weeks, there was a significant reduction between baseline anxiety on the Hospital Anxiety and Depression Scale Anxiety subscale (mean = 14.45, SD = 3.36) 15.42, SD = 3.12) and 8 weeks (mean = (t(55)=2.6, p=.012) and perceived stress measured by the perceived stress scale at baseline (mean = 23.59, SD = 3.99) and 8 weeks (mean = 21.12, SD = 3.09), (t(56)=5.94, p<.001). This study offers preliminary evidence that mHealth Mindfulness Therapy strategies may help caregivers manage the stress and anxiety associated with caregiving.

